# Implementation leadership and implementation climate in context: A single organization intrinsic case study for implementation of digital measurement-based care

**DOI:** 10.1177/26334895241236680

**Published:** 2024-03-28

**Authors:** Marisa Sklar, Mark G. Ehrhart, Nallely Ramirez, Kristine Carandang, Nicolle Kuhn, Ana Day, Gregory A. Aarons, Nathaniel J. Williams

**Affiliations:** 1 Department of Psychiatry, 8784 University of California, San Diego, La Jolla, CA, USA; 2 UC San Diego ACTRI Dissemination and Implementation Science Center, La Jolla, CA, USA; 3 229241 Child and Adolescent Services Research Center, San Diego, CA, USA; 4 Department of Psychology, 6243 University of Central Florida, Orlando, FL, USA; 5 Institute for the Study of Behavioral Health and Addiction, 1791 Boise State University, Boise, ID, USA; 6 Oregon Community Programs, Eugene, OR, USA; 7 School of Social Work, 1791 Boise State University, Boise, ID, USA

**Keywords:** implementation leadership, implementation climate, implementation, case study, organizational context, alignment

## Abstract

**Background:**

Although studies have demonstrated that implementation leadership and climate are important constructs in predicting evidence-based practice (EBP) implementation, concrete descriptions of how they operate during organizational implementation efforts are lacking. This case study fills that gap through an in-depth description of an organization with effective implementation leadership that successfully built a strong implementation climate. This case study provides an illustration of implementation leadership and climate in tangible, replicable terms to assist managers, practitioners, and researchers in addressing the organizational context in their own implementation projects.

**Method:**

A single organization, intrinsic case study was employed to paint a multifaceted picture of how one organization leveraged implementation leadership to strengthen a climate for the successful implementation of digital measurement-based care. The case was drawn from a cluster-randomized trial designed to test the effects of a leadership-focused implementation strategy on youth-level fidelity and clinical outcomes of digital measurement-based care. Following the completion of the trial, case study activities commenced. Descriptive summaries of multiple data sources (including quantitative data on implementation leadership and climate, coaching call and organizational alignment meeting recordings and notes, and development plans) were produced and revised iteratively until consensus was reached. Leadership actions were analyzed for corresponding dimensions of implementation leadership and climate.

**Results:**

Specific actions organizational leaders took, as well as the timing specific strategies were enacted, to create a climate for implementation are presented, along with lessons learned from this experience.

**Conclusion:**

This case study offers concrete steps organizational leaders took to create a consistent and aligned message that the implementation of a specific EBP was a top priority in the agency. The general approach taken to create an implementation climate provides several lessons for leaders, especially for EBPs that have broad implications across an organization.

## Plain Language Summary

Using treatments with known positive impact in community-based mental health programs is challenging. Many studies suggest leaders of these programs can help. Similarly, certain features of community-based programs can also be helpful. This case study of an outpatient mental health clinic provides rich descriptions of actions leaders took that shaped the environment in their program and helped improve the use of a treatment with known positive impact. This case study can serve as a practical guide for leaders to reference when aiming to improve the use of treatments with known impact in their own programs.

## Introduction

Implementation leadership and implementation climate are noteworthy constructs when considering the influence of organizational context on implementation (Aarons et al., 2014a). Implementation leadership concerns the behaviors leaders engage in that emphasize and support evidence-based practice (EBP) implementation (Aarons et al., 2014b). Aarons et al. (2014b) included four types of leader behavior in their initial model of implementation leadership. These dimensions were the following: (a) *proactive*—anticipatory steps taken to problem-solve the implementation process, (b) *knowledgeable*—understanding when and how to use the EBP and effectively communicating this to employees, (c) *supportive*—recognizing, aiding, and reinforcing employee efforts to use the EBP, and (d) *perseverant*—steadfastly attending to implementation through difficult and smooth times and openly addressing challenges. Implementation climate addresses staff members’ shared perceptions regarding the extent to which the policies, practices, and procedures within their organization or unit are aligned with the goal of EBP implementation (Ehrhart et al., 2014). Ehrhart et al. (2014) included six dimensions of implementation climate in their model: (a) *focus*—attention to, and prioritization of, the implementation effort relative to other organizational priorities, (b) *educational support*—the provision and availability of educational resources and trainings for the EBP, (c) *rewards*—the provision of financial incentives for using the EBP, (d) *recognition*—acknowledgment and/or elevated status for EBP use, (e) *selection for EBP*—the selection/hiring of new staff members based upon their experience or expertise with the EBP, and (f) *selection for openness*—the selection/hiring of new staff members based upon their willingness and adaptability to use new types of interventions.

Research has demonstrated the link between implementation leadership, implementation climate, and EBP implementation (Williams et al., 2018, 2022c). A longitudinal study of mental health clinics illustrated that increases in clinic-level implementation leadership over 5 years were associated with increases in implementation climate, which were subsequently associated with providers’ self-reported EBP use (Williams et al., 2020). Other research has shown that implementation climate is associated with higher therapist fidelity to cognitive-behavioral therapy with patients (Williams et al., 2022a), higher fidelity to a complex EBP for autism in schools (Williams et al., 2022b), more effective implementation and reach in the context of medication management programs in pharmacies (Turner et al., 2018), and increased intensity of EBP coverage in clinical supervision (Pullmann et al., 2018).

Although studies have demonstrated that implementation leadership and climate are important constructs in predicting EBP implementation, rich, concrete descriptions of how these concepts operate in specific and tangible ways in organizational implementation efforts are lacking. This case study is designed to fill that gap by providing an in-depth description of an organization that was able to enact effective implementation leadership and build a strong implementation climate. By explicitly sharing steps taken by agency leaders to mold an organizational climate to support effective implementation, this case study de-mystifies the constructs of implementation leadership and climate to serve others in addressing the organizational context in their own implementation projects.

## Method

### Study Design

Given the goal of rich and deep description, a single organization, intrinsic case study was employed (Crowe et al., 2011). The case was drawn from a cluster-randomized hybrid type III effectiveness-implementation trial designed to test the effects of the Leadership and Organizational Change for Implementation (LOCI; Aarons et al., 2015, 2017) strategy on youth-level fidelity and clinical outcomes of digital measurement-based care (R01MH119127). In total, 21 outpatient behavioral health clinics that served youth participated in the trial and 11 of these were randomly assigned to participate in LOCI. All trial participants provided informed consent, and this trial was approved by the Boise State University Institutional Review Board.

Following the intrinsic case study approach, a single organization was selected for analysis based on its perceived uniqueness. Specifically, after the conclusion of the LOCI strategy, the research team viewed this organization as the most successful in identifying and enacting strategies to promote implementation leadership and implementation climate. In discussions between the research team and organizational leadership following completion of LOCI, leaders in the organization similarly indicated they were successful in adopting strategies to promote implementation, thus demonstrating concordance between researcher and participant perspectives.

The intrinsic case study approach was favored over other designs (such as an instrumental case study) as it is well suited for in-depth exploration of substantively interesting and unique phenomena; in this case, a compelling, concrete example of implementation leadership and climate within an organization that optimally supported these targets (Crowe et al., 2011). The epistemological approach was interpretive in that it attended to multiple perspectives of the case and aimed to convey the experience of this organization in a manner that enables readers to “recognize essential similarities to cases of interest to them” (Stake et al., 1978, p. 7), aiding generalizations to their own situations. The nature of the inquiry was thus purposefully broad, with the goal of exploring a phenomenon and providing a narrative to inform others working toward implementation (Yin, 2018). Reflexive participant collaboration (Motulsky, 2021) was sought throughout, such that leaders from the organization and researchers collaborated on analysis, interpretation, and presentation of findings.

### Measurement-Based Care and the Outcomes Questionnaire Analyst

The EBP being implemented in this study was a digital measurement-based care system called the Outcomes Questionnaire-Analyst (OQ-A). The OQ-A is a commercially available, web-based application that incorporates well-established psychometrically tested measures, automatic electronic scoring, and generation of feedback reports for each client based on expected recovery curves produced from big data algorithms (Dunn et al., 2005; Lambert, 2012; Lambert et al., 2018). Organizations had access to the Youth Outcomes Questionnaire 30.2 (Dunn et al., 2005), which assesses youth symptoms and functioning in multiple domains (OQ Measures, 2016; OQ Measures, 2018), and the treatment support measure for youth and caregivers (Harmon et al., 2007). Clients complete standardized measures via electronic tablet or through a texted link and feedback reports are available almost immediately. Developers of the OQ-A system encourage providers to share feedback with clients and to discuss its implications for treatment (Lambert, 2012).

Providers who worked with youth, clinical supervisors, and leaders from participating organizations were invited to attend three trainings on the OQ-A hosted by the OQ-A purveyor organization (PhD-level psychologist), including an initial 1-day in-person training and two live, 1-h, web-based booster trainings. Trainings provided information on the clinical utility of the OQ-A, step-by-step instructions for administering questionnaires and accessing feedback reports, interpretation of metrics, and best practices for introducing the measures to youth and caregivers and using the information to inform clinical care. Trainings were recorded and accessible to participants. Additionally, each provider organization received technical assistance from the OQ-A purveyor, including an assigned representative to provide ongoing technical assistance, an online library of training videos, and web-based site-specific training as needed.

### Implementation Strategy

**Figure 1:**
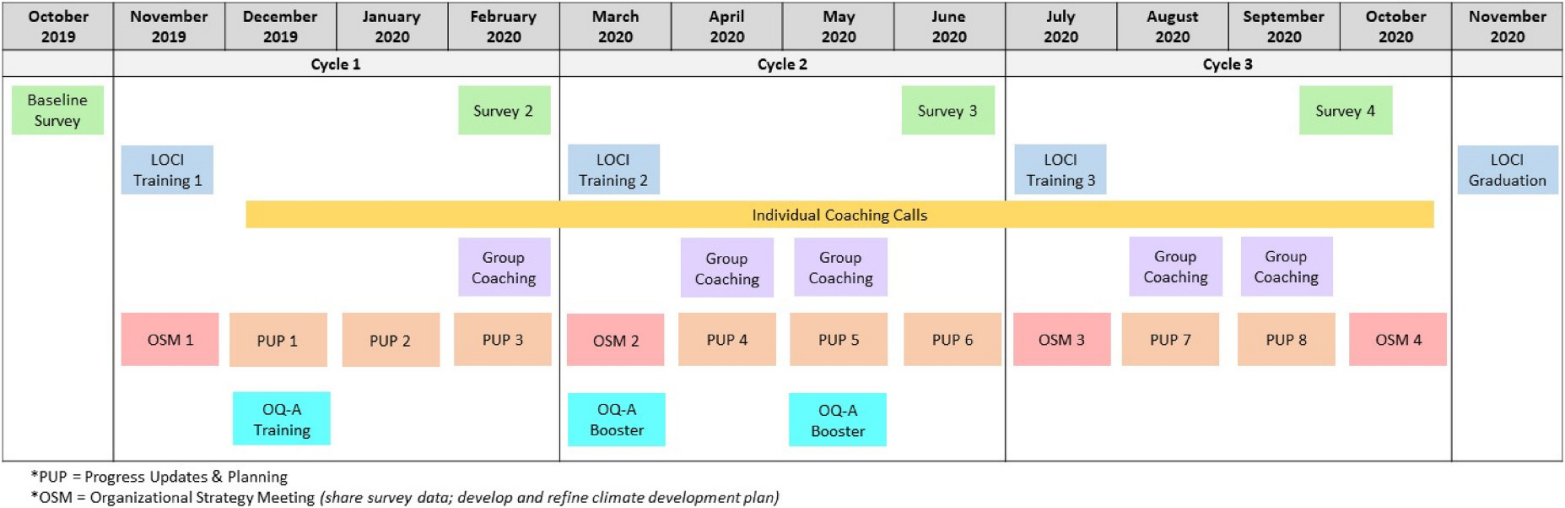
Timeline of Trial Activities

In the trial from which this case study was drawn, 11 clinics from the participating organizations, including the target organization for this case study, were randomized to engage in LOCI, delivered by its developers (GAA and MGE) and a team of experienced and trained support staff (KC, NR, MS, and NJW). LOCI (2022) is a multifaceted implementation strategy that aims to improve general and strategic implementation leadership and climate within organizations to support implementation of EBPs with fidelity (Aarons et al., 2015, 2017). LOCI draws upon two leadership approaches, the full-range leadership model (Avolio et al., 1999; Bass & Avolio, 1990) and implementation leadership (Aarons et al., 2014c), as well as theories on organizational implementation climate and climate/culture embedding mechanisms (Ehrhart et al., 2014; Klein & Sorra, 1996; Schein, 2010). To achieve its aims, LOCI engages participants in the following interrelated components: data and feedback, leadership development trainings, coaching, and organizational alignment. LOCI utilizes repeated cycles wherein data on leadership and climate is collected and shared with participants to assess progress over time, participants attend leadership development trainings to learn about full-range and implementation leadership and implementation climate, and leadership and climate development plans are crafted to outline actions organizational leaders commit to taking to facilitate implementation of the EBP. See Figure 1 for LOCI cycles and activities over time. First-level leaders (i.e., those who supervise direct service providers) engage in trainings and individual (∼15 min in duration) and group coaching calls. Organizational executive leaders engage in the organizational alignment component that includes organizational strategy meetings (OSMs) and progress update and planning (PUP) meetings. During OSMs, data on climate are shared with both organizational executive leadership and first-level leaders, and goals are outlined to promote a climate for implementation within the organization. PUP meetings review progress the organization has made toward these goals.

### Oregon Community Programs

This single organization intrinsic case study focuses on Oregon Community Programs (*About Oregon Community Programs*, 2022) a privately held, non-profit mental health organization located in Eugene, Oregon, USA. OCP has provided research-based treatment and prevention services for children, youth, and families since 1983. OCP initially focused on community implementation of EBPs developed by the Oregon Social Learning Center, an NIH-funded, non-profit research group focused on supporting positive family development and social learning. Over time, OCP expanded services to include outpatient behavioral health treatment to children and families of the whole community including family therapy, individual therapy, skills training, case management, psychiatry, and crisis support. At the time of LOCI participation, OCP reported having served 467 youth in the last year and had 20.5 FTE clinicians. On average, therapists had a caseload of 25 clients.

OCP has an explicit focus on providing high-quality care through implementation of EBPs. All services provided are informed by EBP with extensive research and training to support their staff. Their mission is “to support the use of EBP treatment for children and families with training, consulting, and outreach services to promote a positive family life” (*Welcome to Oregon Community Programs*, 2022, p. slide 1). When initially contacted about participating in LOCI for OQ-A implementation, OCP leadership reported having recently attempted to implement a different measurement-based care system with suboptimal results. Although eager to implement measurement-based care, OCP leadership would only agree to proceed if the evidence for the OQ-A system suggested it was an improvement upon this prior system.

### Data and Analysis/Procedures

Multiple data sources were analyzed to better understand OCP leaders’ enactment of implementation leadership behaviors/actions to generate an implementation climate for OQ-A: Quantitative results on implementation leadership and climate, participation logs detailing first-level leader and executive leader participation in trial activities, leadership and climate development plans, coaching call notes, coaching call recordings, and organizational alignment meeting recordings.

#### Implementation Leadership

The Implementation Leadership Scale (ILS) (Aarons et al., 2014c) is a 12-item measure of unit-level leadership for EBP implementation with excellent reliability and convergent and discriminant validity (Aarons et al., 2016; Finn et al., 2016). The four ILS subscales each consist of three items and are Proactive (α = .95), Knowledgeable (α = .96), Supportive (α = .95), and Perseverant (α = .96), which are combined into a total score (α = .98). Each item is scored on a 5-point Likert-type scale (0 = *not at all*, 4 = *to a very great extent*).

#### Implementation Climate

The Implementation Climate Scale (ICS) (Ehrhart et al., 2014) assesses employees’ shared perceptions of the policies, practices, procedures, and behaviors that are expected, supported, and rewarded to facilitate effective EBP implementation. All items are scored on a 5-point Likert-type scale (0 = *not at all* to 4 = *to a very great extent*). The ICS has excellent internal consistency and convergent and discriminant validity (Ehrhart et al., 2019, 2021). The ICS has an overall Cronbach's alpha of .91 (18 items, three items on each subscale). The six subscales are Focus on EBP (α = .91), Educational Support for EBP (α = .84), Recognition for EBP (α = .88), Rewards for EBP (α = .81), Selection for EBP (α = .89), and Selection for Openness (α = .91).

#### Data Analysis

Consistent with the interpretive epistemological approach for this intrinsic case study, data were analyzed in a manner that facilitated flexible and descriptive detailing of the phenomena present (Crowe et al., 2011). Descriptive statistics were used to summarize quantitative data. Quantitative and qualitative data were analyzed and reviewed iteratively among researchers and behavioral health organization partners. Descriptive summaries of data were drafted and revised until agreement between all partners was reached. Specifically, coaching call recordings and organizational alignment meeting recordings were reviewed retrospectively by author MS for concordance with, and expansion of, participation logs data and leadership and climate development plan content. Several authors (MGE, MS, NJW) reviewed content of leadership and climate development plans and labeled descriptions of leader strategies with corresponding dimensions of implementation leadership and climate. Detailed descriptions of leadership actions were reviewed by all authors iteratively, and revised when appropriate, until consensus was reached. This approach was favored over an approach involving the creation of codes to represent emergent themes. In describing case study methodology, Stake asserted that while “themes and hypotheses may be important … they remain subordinate to the understanding of the case” (Stake et al., 1978, p. 7), further asserting that analysis should favor descriptions that are complex and holistic, and a narrative, verbatim writing style. The authors aimed to approach this case study in a manner consistent with Stake's assertions.

## Results

Three leaders at OCP directly engaged in LOCI to develop strategies that supported implementation of OQ-A. One of these leaders (clinical project manager) engaged in LOCI as a first-level leader, and the other two leaders (executive director and outpatient services program director) engaged in LOCI as executive leaders. The specific strategies these leaders took to support implementation of OQ-A within OCP are presented in accordance with the cycle within which the strategies were initially established and enacted. See Table 1 for a list of these strategies.

**Table 1:** Summary of Strategies Used by Oregon Community Programs (OCP) Leadership and Staff for Implementation of the Outcomes Questionnaire-Analyst (OQ-A), and Corresponding Dimensions of Implementation Leadership and/or Climate

Dimension	Goal	Action steps	Cycle established
Focus and Proactive	Communicate the importance and prioritization of OQ-A to all staff.	Describe expectations for OQ-A implementation start date to all staff.	1
		Inform staff of the specific clients for whom the OQ-A will be used for.	1
		Role model their own use of OQ-A	1
Supportive and Educational Support	Increase availability of first-level leader support across all OCP programs.	Attend one clinical meeting per program per month.	1
		Schedule office hours dedicated to discussing implementation with staff.	1
		Email the availability and purpose of office hours to staff to increase awareness and use of this resource.	1
Proactive	Prepare staff for implementation of OQ-A workflow.	Meet with program managers to solicit feedback on workflow, and determine which outcome measures used prior to OQ-A implementation would continue to be used (and which would be discontinued).	1
		Develop first draft of workflow.	1
		Schedule meeting with staff immediately after OQ-A training to get feedback on workflow draft.	1
		Revise workflow draft based on feedback.	1
Educational Support	Increase sustained educational support for OQ-A	Develop document detailing an overview of the OQ-A training.	1
		Develop a shorter OQ-A fact sheet.	1
		Share detailed overview and shorter fact sheet with clinical staff for feedback.	1
		Revise detailed overview and shorter fact sheet based upon feedback.	1
Rewards and Recognition	Develop short- and long-term/sustainable systems for recognizing and rewarding OQ-A implementation efforts.	Develop a raffle for staff who text clients the link to complete assessments in the OQ-A system.	2
		Develop incentive for 70% of reports being viewed within 1 week of assessment administration.	2
		Facilitate group/clinic-level goal setting and reward mechanisms.	2
Supportive and Proactive	Provide program managers with resources to integrate OQ-A into supervision.	Add OQ-A as a standing agenda item during clinical supervision meetings.	2
		Provide managers with copies of OQ-A report steps at manager's meeting.	2
		Engage in group discussion on using OQ-A in supervision at in-person manager's meeting.	2
		Update supervision form to include OQ-A.	2
		Ask clinicians to include insights from OQ-A within case presentation during group and individual supervisions.	1
Focus and Selection for OQ-A	Integrate OQ-A into OCP policies, practices, and procedures.	Create script for reception to follow that prompts clinicians to review client reports in OQ-A	1
		Include OQ-A workflow, training overview, fact sheet, and link to the OQ-A training video to new employee binder.	1
		Add OQ-A to job descriptions, mission/values, and performance evaluations.	2
		Add OQ-A to client intake forms.	3
Rewards and Recognition	Communicate OQ-A success stories across OCP.	Ask program managers to identify consistent OQ-A users/OQ-A champions.	2
		Solicit OQ-A success stories from the identified consistent OQ-A users/OQ-A champions.	2
		Incentivize the sharing of success stories by offering rewards.	2
		Share OQ-A success stories across OCP via email.	2
Focus	Use data from OQ-A to support ongoing implementation.	Download data on weekly basis to generate reports.	2
		Analyze trends in data over time.	2
		Send monthly email to all staff summarizing OQ-A data that includes number of assessments completed, trends in the data, and how many client reports were reviewed by clinical care staff.	2
		Discuss critical items from data in weekly managers’ meeting.	2
		Integrate data into client-specific documentation (mental health assessments, service plans, treatment documentation, prior authorizations) if/when appropriate.	2
Focus and Proactive	Manualize OQ-A processes to facilitate long-term sustainment.	Develop guide that outlines how data from OQ-A should be analyzed, presented, and disseminated to staff.	3
		Outline process for integrating OQ-A data into performance evaluations.	3
		Protocolize process for onboarding new staff on OQ-A.	3

### Cycle 1

**Figure 2:**
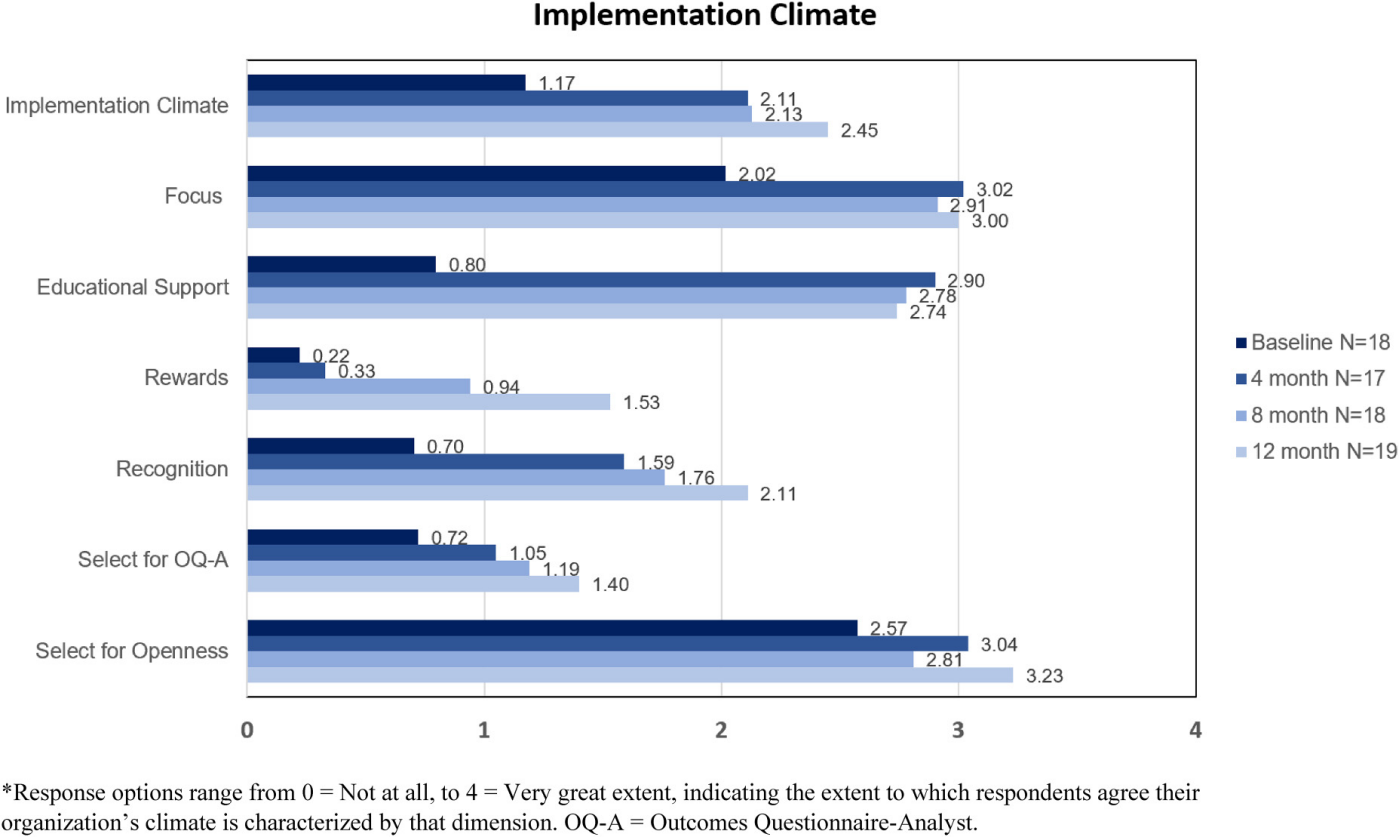
Provider Ratings of Implementation Climate for OQ-A Over Time at Oregon Community Programs

**Figure 3:**
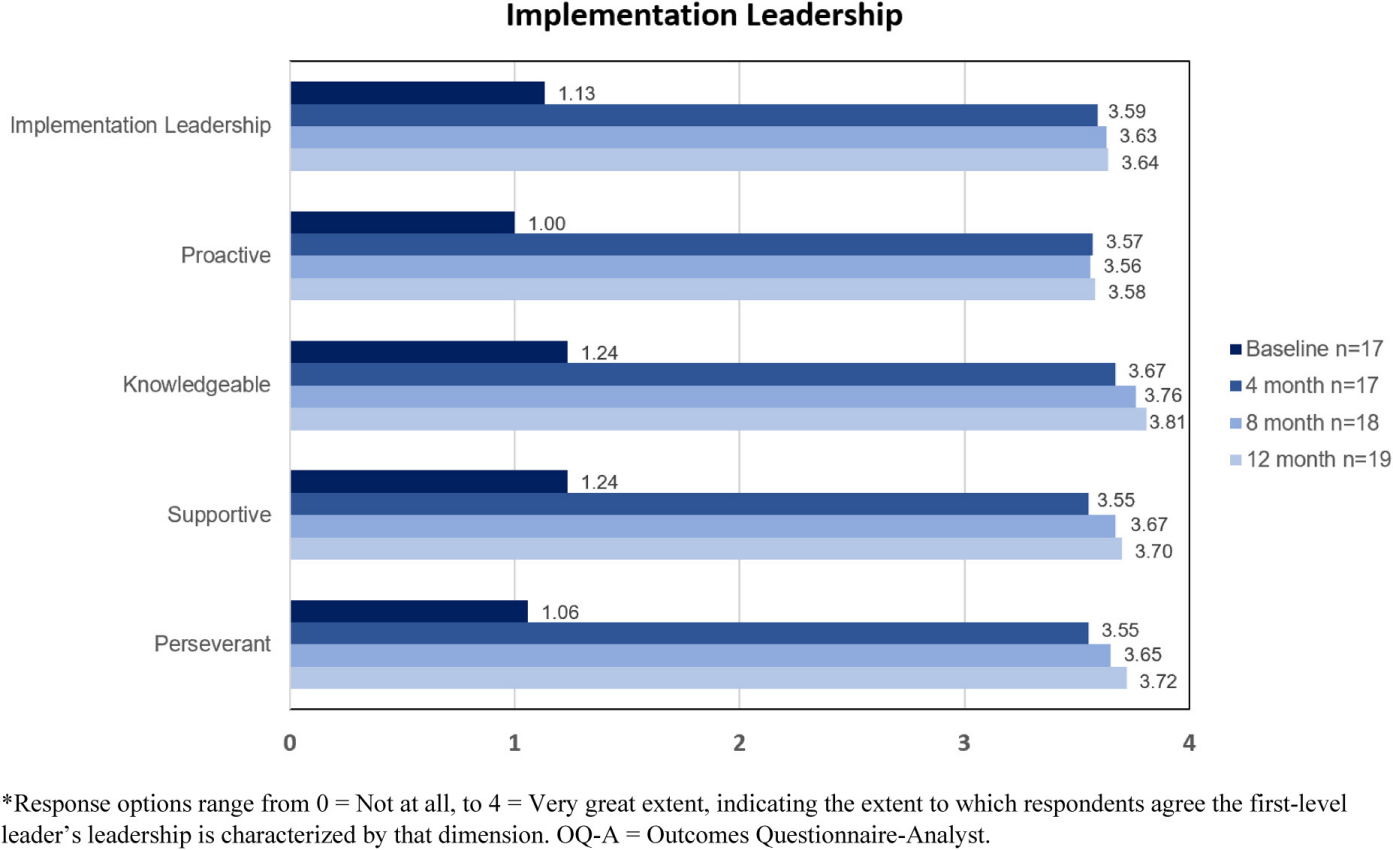
Provider Ratings of Implementation Leadership for OQ-A Over Time at Oregon Community Programs

At the start of the first cycle of OCP's engagement in LOCI, leaders were presented with results from baseline surveys that assessed general and implementation leadership of the first-level leader, as well as the implementation climate at OCP, using the abovementioned ILS and ICS instruments. See Figures 2 and 3 for provider ratings of implementation leadership and climate at OCP over time. Informed by these data, the first-level leader drafted a leadership development plan targeting dimensions of implementation leadership that would foster a *proactive* and *supportive* approach to implementation. Specific goals to enhance supportive leadership included increasing availability to staff members across treatment programs by attending one clinical meeting per program per month and by scheduling regular office hours. Plans to increase proactive leadership included soliciting feedback from managers on the early stages of the OQ-A implementation, drafting a workflow for integrating OQ-A into standard operating procedures and meeting with staff members to get their feedback on the workflow draft.

*Response options range from 0 = *not at all* to 4 = *very great extent*, indicating the extent to which respondents agree their organization's climate is characterized by that dimension. OQ-A = Outcomes Questionnaire-Analyst.

*Response options range from 0 = *not at all* to 4 = *very great extent*, indicating the extent to which respondents agree the first-level leader's leadership is characterized by that dimension. OQ-A = Outcomes Questionnaire-Analyst.

**Figure 4:**
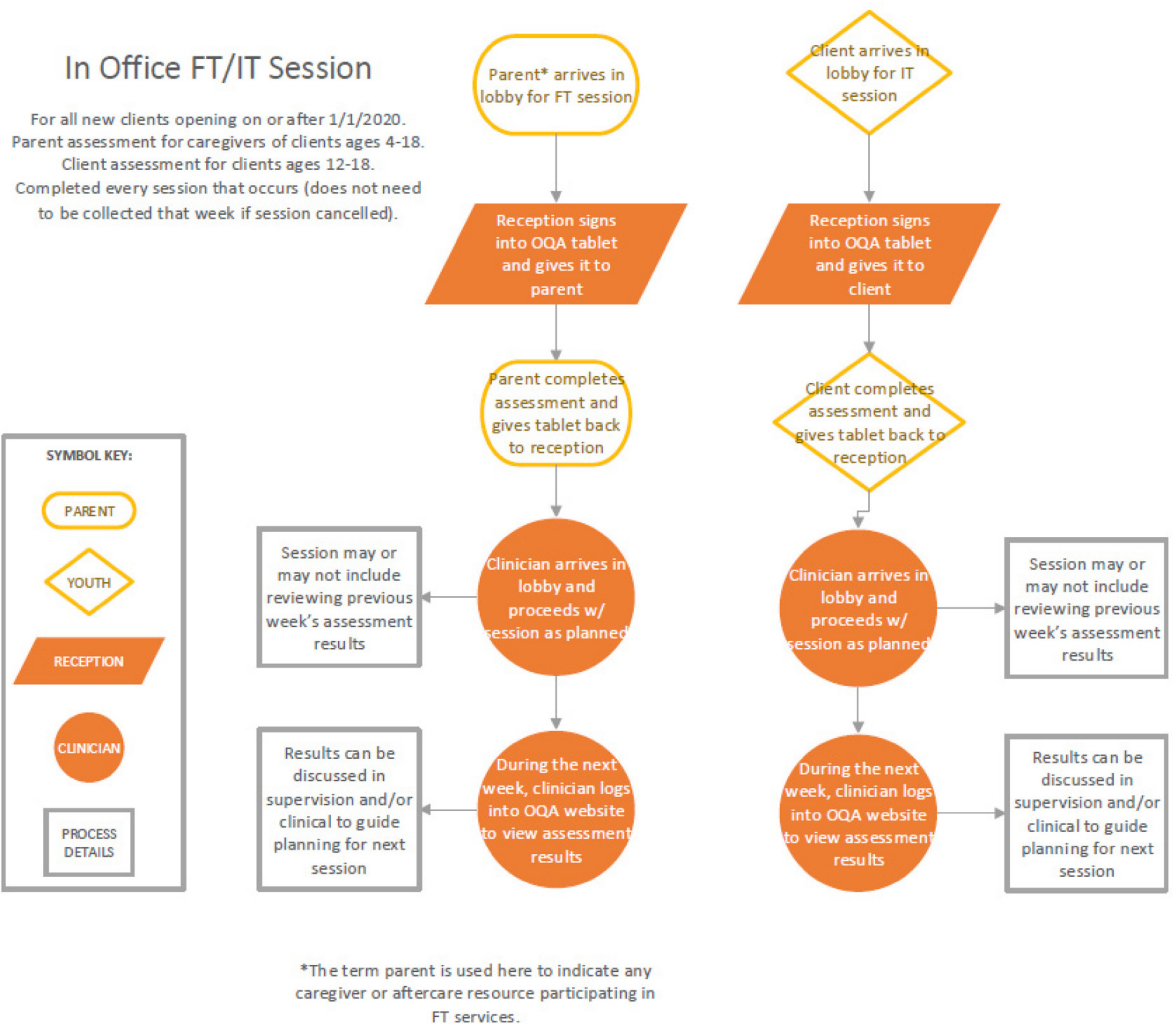
Workflow for OQ-A Implementation for Family Therapist and Individual Therapist Services

As Cycle 1 progressed, the first-level leader continued to address proactive leadership by meeting with executive leadership and program directors to review a first draft of an OQ-A implementation workflow and determine which outcome measures used by the clinic prior to OQ-A implementation would continue to be used in conjunction with OQ-A (and which would be discontinued). See Figure 4 for workflow. Workflow drafts were shared with clinical staff for feedback and changes were made accordingly. Strengthening the implementation climate dimension of *educational support*, a document providing an overview of the OQ-A training that providers attended and a shorter OQ-A fact sheet were developed and shared with clinical staff for feedback. The first-level leader also joined monthly clinical meetings to strengthen the implementation climate dimension of *focus* by leading ongoing discussions of OQ-A implementation and integration with clinical care. The first-level leader's stated intention in joining these meetings was to give clinical staff “space to talk through how OQ-A implementation [was] going, answer any questions, and share experiences.” Weekly office hours were initiated by the first-level leader to provide additional *educational support* to clinical care staff and problem-solve barriers to OQ-A implementation. Consistent with *perseverant* leadership, OCP leadership supported the generation of solutions to enhance measurement completion rates across clientele. For example, if a client did not complete a measure at check-in, reception staff wanted a discrete method for informing the clinicians that the measure had not yet been completed by the client and should be followed up with. Consequently, reception staff created a script to prompt clinicians to review client reports in the OQ-A. Demonstrative of both *proactive* leadership and a *focus* on OQ-A implementation, the first-level leader enacted several strategies to facilitate OQ-A sustainment in the context of potential turnover and onboarding of new staff. Specific actions include adding the OQ-A workflow, OQ-A training overview, OQ-A fact sheet, and a link to the OQ-A training video to new employee binders.

In the first cycle, executive leadership at OCP created an initial climate development plan to target specific climate dimensions identified as priorities following the baseline organizational survey. Goals on the climate development plan were centered on initial implementation of the OQ-A. To *proactively* anticipate questions and clarify expectations regarding implementation, leadership communicated with staff the start date for implementation, and the clients for whom the OQ-A would be used. In line with the ICS *focus* dimension, executive leaders committed to communicating the importance of the OQ-A through role modeling their own use of the system and making OQ-A a standing agenda item during clinical supervision meetings. To further reinforce a *focus* on OQ-A implementation, clinicians were asked to include insights from OQ-A within case presentation at group and individual supervisions. Additionally, executive leaders committed to *rewarding* and *recognizing* implementation by developing an incentivization process for use of OQ-A. Specifically, executive leaders began exploring the possibility of developing a bonus system around the use of OQ-A and began reinforcing efforts to use OQ-A in the interim through small rewards, such as chocolate and coffee cards. To create an aligned *focus* on implementation across levels of leadership, executive leadership committed to reinforcing the messages of the first-level leader during group meetings, and to describe the organization's commitment to OQ-A during all-staff meetings.

### Cycle 2

At the start of the second cycle of OCP's engagement in LOCI, leaders were presented with results from the second survey. The first-level leader refined their leadership development plan based upon these results, adding goals to increase *recognition* and *rewards* for OQ-A implementation. To continue short-term recognition and rewards, she committed to implementing a raffle for staff who texted clients a link to the OQ-A system for ongoing measurement. She also planned to develop an incentive for staff who viewed a minimum of 70% of their reports in OQ-A within 1 week. In line with the implementation leadership dimensions of *supportive* and *proactive* leadership, by anticipating potential barriers and offering solutions, this leader also added a goal to provide supervisors with resources so they could better integrate OQ-A into clinical supervision. Specific resources she committed included providing managers with copies of a step-by-step process managers could follow to access reports in OQ-A at an upcoming managers’ meeting, and leading a group discussion on how to use the OQ-A in supervision. The final goals that were added were to better integrate OQ-A in OCP job descriptions, OCP mission/values, supervision forms, and performance evaluations, targeting implementation climate dimensions of *focus* and *selection for OQ-A*.

As Cycle 2 progressed, the first-level leader transitioned from the position of part-time clinical project manager and part-time provider of clinical services, to full-time clinical project manager focused exclusively on implementation and sustainment of OQ-A. She reported seeing an increase in use of the OQ-A with a majority of clinicians using it, and she supported managers’ running of reports within the system and integrating these reports into clinical supervision. This leader instituted the raffle and began emailing an OQ-A “Tip of the Week” to staff. Success stories were solicited, and OQ-A champions were identified (*recognition*). Consistent with the climate dimension of *focus* on OQ-A, the first-level leader began emailing monthly OQ-A results that summarized the number of self-report and parent-report assessments completed during the month, trends in the data, and how many client reports were reviewed by clinical care staff. Additionally, one administrative staff member was *recognized* as an OQ-A “co-champion” and assumed responsibility for entering clients into the system, assisting other administrative staff to use OQ-A, generating reports by downloading the data on a weekly basis, tracking and analyzing trends in data over time, and sharing data and reports with the first-level leader. Similar to the first-level leader, this “co-champion” also sought support from OQ-A representatives to learn more about generating reports. Critical items on the OQ-A were being discussed in weekly managers’ meeting, and data were also being incorporated into client-specific documentation, demonstrating a *focus* on OQ-A implementation. For example, OQ-A data were referenced in mental health assessments, service plans, and treatment documentation, and was being used to justify higher levels of care with payers.

In this second cycle, executive leadership at OCP similarly set new goals that targeted *rewards* and *recognition* for OQ-A implementation. Executive leaders developed group-level incentives and worked with technical support from the OQ-A team to create “groups” within the system to aggregate use per clinic/program. Executive leaders agreed to offer $5 incentives to staff members who submitted success stories and committed to designing and distributing a visual representation of the OQ-A success stories to recognize staff members’ progress. Additionally, the decision to make the first-level leader's project manager role full-time was indicative of the executive leadership's *support* for the implementation project.

### Cycle 3

At the start of the third cycle, results from the third survey were shared with the OCP leadership team. No new additional goals were outlined in the first-level leader's plan, though she noted having further integrated OQ-A by including the OQ assessment in all new client intake packets, strengthening OCP's *focus* on OQ-A implementation. Consistent with the implementation leadership dimension of *proactive* leadership, this first-level leader shared plans for an upcoming extended leave of absence and focused her efforts on manualizing implementation processes (e.g., processes that outlined how data from OQ-A should be presented and disseminated to staff across OCP, how OQ-A data should be integrated into performance evaluations, etc.) and identifying appropriate staff to take on her responsibility of supporting OQ-A implementation in her upcoming absence. She described advancing group/clinic-level reporting, goal setting, and incentivizing—further enhancing *rewards* and *recognition* for implementation. Specifically, each group/clinic outlined a goal they wanted to work toward over a 1-month period. One clinic opted to work toward having at least 70% of their clients complete an assessment in the OQ-A. Another clinic opted to work toward having at least 79% of assessment reports viewed by clinical care staff within 1 week following assessment completion. For groups/clinics that reached their outlined goal, $15 gift cards were administered to each staff member within that group/clinic that could be combined and used for buying supplies/conducting group/clinic activities. A more detailed process for onboarding new staff on OQ-A was manualized, further enhancing the implementation climate dimensions of *focus* and *selection* for OQ-A implementation. Two staff members were identified and began training to take over the first-level leader's role.

During this third cycle, executive leadership similarly opted to continue working toward already established goals on their climate development plan and not add additional goals. Specifically, a financial reward system was established so that top performers in the OQ-A system could receive annual bonuses. Performance evaluations were also reformatted to include OQ-A metrics. Both of these actions further enhance the implementation climate dimensions of *focus* and *rewards/recognition* for OQ-A implementation.

## Discussion

One of the primary goals of this case study was to describe an example of an organization's successful fostering of implementation leadership and climate. Different readers may find different aspects of this case study valuable for their own research and practice, in line with Stake's (1978) perspective of case studies. Additionally, we wanted to highlight a few key lessons learned from this experience that encompass the constructs of implementation leadership and climate. Following the presentation of lessons learned, we acknowledge limitations and offer future directions.

### Lessons Learned

One factor that influenced OCP's success in implementation was their prioritization and responsiveness to the process. Having favorable attitudes toward EBPs has been shown to predict successful implementation (Locke et al., 2019a; Nelson et al., 2012; Nelson & Steele, 2007; Reding et al., 2014). OCP's longstanding history of valuing research and the objective demonstration of treatment effectiveness was evident in the authentic signals and actions that leaders took to promote implementation—and these actions can be replicated elsewhere. Prior to engaging in the larger trial from which this case study was drawn, OCP had attempted to implement a different system for measurement-based care that proved to be unwieldy and unhelpful; consequently, before deciding to participate in this trial, OCP executive leadership appraised the OQ-A system to ensure it would be an improvement upon the prior system and that it would fit their organization (Klein & Sorra, 1996). Specifically, they wanted a system that was highly researched, evidence-based, and capable of illustrating program outcomes in a compelling manner for rate setting with insurance funders. They also wanted to ensure the system was reasonably simple to use, applicable to their client population (caregiver and youth), and affordable for use beyond the trial. This response to prior implementation failure, of persistently searching for a better alternative and committing to its success, reflects the dimension of perseverant leadership.

Another key signal of leadership's support, prioritization, and responsiveness to implementation was the assignment of a single staff person to oversee the implementation process. By the second cycle, the first-level leader had transitioned from part-time to full-time clinical project manager with a primary focus on leading the OQ-A implementation. This focused allocation of resources created a natural organizational champion with dedicated time to promote the OQ-A. Having a single individual fully focused on the implementation process had wide-ranging implications and was a recurring theme in conversations with the agency and among the research team for why this agency was so successful in the implementation. OCP executive leaders’ focused allocation of resources to implementation should also be recognized. In addition to protecting the first-level leaders’ time to focus on leading the implementation, executive leaders provided numerous incentives for OQ-A implementation, and incurred costs related to staff time for OQ-A training and support. OCP leaders expressed hopes that initial investments in this implementation would decrease future costs by streamlining clinical care. Furthermore, OCP executive leaders envision a future of managed care connecting to value-based payment for which measurement-based care with OQ-A can be an advantage in contracting.

Lesson #1: Having strong, authentic support, and responsive actions from upper-level leadership from the beginning and throughout the implementation process can play a critical role in implementation success.

A second factor related to OCP's success was that the first-level leader was not solely responsible for the success of the project, but leaders across the agency similarly enacted strategies to propel forward OQ-A implementation. To be clear, having one leader who was the point person on the project was important, but only having that could have been problematic. Our case study description included many examples of other leaders in the agency supporting OQ-A and its implementation. For instance, one of the first steps that OCP took was to integrate OQ-A into the workflow of the agency. In order for that integration to take place, leaders from the administrative and clinical departments had to be willing to make adjustments to their current processes. Clinical leaders had to make OQ-A a standing item in their clinical supervision, in addition to using the information from the OQ-A in their regular clinical group and individual supervision meetings. Executive leaders communicated their support for OQ-A in staff meetings and provided resources to incentivize OQ-A use. Other leaders filled roles, such as the “co-champion” for OQ-A, and new staff had to take on some of the OQ-A-related responsibilities when the first-level leader went on leave. The importance of shared leadership responsibility for implementation in this case study supports the hypothesis that “distributed leadership” models, in which leaders with different types of expertise and different roles collaborate to support implementation, are important for successful implementation of complex practices (Locke et al., 2019b). This case study also provides a rich example of alignment across leadership levels, which prior research has described as potentially important for implementation success (Aarons et al., 2014b; Ehrhart & Aarons, 2022; Lundmark et al., 2021). The actions of a single leader may be inadequate for successful implementation; rather, the congruence of actions of multiple leaders throughout an organization is optimal. This alignment in actions across multiple leaders played a critical role in the success of the OQ-A implementation for OCP.

Lesson #2: Although having a single leader in charge of the implementation is important, other leaders throughout the agency must acknowledge their role in the implementation process, and take action to contribute to implementation effectiveness.

A third lesson learned from this case study is related to the breadth of changes that may be necessary for implementation success. The implementation literature has highlighted how practices vary in complexity, from relatively simple actions taken by only a single or small percentage of providers, to more complex, coordinated sets of actions involving multiple staff and functions (Amodeo et al., 2011; Williams et al., 2022b). Particularly in the latter case, the broader the implications of the new practice, the broader the implementation strategy should be in terms of aligning the policies, practices, and procedures in the agency, and the more critical the development of an implementation climate will be. In this case study, the OQ-A system would be considered a complex intervention due to the number of staff involved in its use and the technological as well as clinical implications; consequently, many strategies were applied to its implementation. See Table 1 for a list of these strategies. Although all of these strategies may seem like a lot, it is the consistent messaging across all of them that OQ-A is a priority that creates the implementation climate. With regard to strategic climates, such as implementation climate, it is the convergence of multiple strategies to promote strategic goals throughout an organization that results in the development of a positive and strong climate (Aarons et al., 2014b).

Lesson #3: The more complex a new practice is, the more critical it is to create an implementation climate in the agency by integrating it into a wide swath of the organizational policies, practices, and procedures.

A final lesson from this case study is related to sustainment. To implement well, organizational leaders should consider sustainment from the start. OCP considered sustainment at the outset and as part of the creation of a strong implementation climate, created a variety of structures to ensure that OQ-A would become an integral part of the organization even after trial completion. Examples include the integration of OQ-A into job descriptions and onboarding processes for new staff, including OQ-A in all new client intake packets, reformatting performance evaluations to include OQ-A metrics, adding OQ-A to paperwork for clinical supervision, manualizing the use and reporting of OQ-A, and adding OQ-A data into reports to payers. In each of these cases, the changes not only emphasized the importance of implementation and helped to create the implementation climate, but the changes were so integrated into the organizational processes that they would be difficult to ignore or avoid. Especially when such integration occurs across so many different processes, the new practice becomes the typical way work gets done in the organization.

Lesson #4: Broadly integrating implementation of the new practice into existing organizational structures strongly increases the likelihood of not only implementation success, but long-term sustainment.

### Limitations

This single organization case study provides a rich description of what idealized implementation leadership and climate look like when done well in a specific clinic; however, it has limitations. The extent to which this clinic's experience may generalize to other clinics or organizations is unknown due to differences in history, policy and funding contexts, size, staffing, and other factors. Nonetheless, a growing number of studies demonstrate that change in organizational leadership and climate are possible and that these changes can improve the clinical outcomes of mental health services (Glisson et al., 2013, 2016; Skar et al., 2022). This case study is also shaped by the researchers’ own perspectives and experiences. To mitigate potential bias, we relied on member checking and co-creation of the report with organizational leaders.

### Future Directions

To address these limitations and expand on this study, more research is needed to better understand the specific settings within which implementation leadership and climate are most impactful in mental health services and what are the limitations on modifying these antecedents to improve implementation. Similarly, research can help elucidate the EBPs for which strong implementation leadership and climate are most critical. It is certainly possible that variability exists with regard to the relevance of specific dimensions of implementation leadership and climate based upon EBP and/or setting. For example, perhaps the implementation climate dimension of rewards is less applicable to substance use settings wherein contributing service to support others’ recovery is strongly emphasized. Research could also be conducted addressing the relative importance of leader support across levels. Although we have argued that all leaders should play their part to contribute to effective implementation, it may be that in some settings and for certain implementation projects, the support of certain levels of leadership may be more critical than others.

## Conclusion

The goal of this case study was to explore concrete and tangible examples of how effective implementation leadership can build a strong implementation climate in agencies. This case study illustrates several specific actions organizational leaders took to create a consistent and aligned message that the implementation of OQ-A was a top priority in the agency. Although actions for implementing other practices elsewhere may differ, the general approach taken to create a climate for OQ-A implementation provides several lessons for leaders, especially for EBPs that have broad implications across an organization. Future research should endeavor to provide rich descriptions of implementation contexts and strategies. In doing so, other organizations may be better guided in their own EBP implementation efforts.
